# FBN30 in wild *Anophelesgambiae* functions as a pathogen recognition molecule against clinically circulating *Plasmodiumfalciparum* in malaria endemic areas in Kenya

**DOI:** 10.1038/s41598-017-09017-3

**Published:** 2017-08-17

**Authors:** Guodong Niu, Genwei Zhang, Caio Franca, Yingjun Cui, Stephen Munga, Yaw Afrane, Jun Li

**Affiliations:** 1 Department of Biological Sciences, Biomolecular Sciences Institute, Florida International University, Miami, Florida 33199 USA; 2 Department of Chemistry and Biochemistry, University of Oklahoma, Norman, Oklahoma 73019 USA; 3 Centre for Global Health Research, Kenya Medical Research Institute, Kisumu, Kenya

## Abstract

Malaria is a worldwide health problem that affects two-thirds of the world population. *Plasmodium* invasion of anopheline mosquitoes is an obligatory step for malaria transmission. However, mosquito-malaria molecular interactions in nature are not clear. A genetic variation within mosquito fibrinogen related-protein 30 (FBN30) was previously identified to be associated with *Plasmodium falciparum* infection in natural *Anopheles gambiae* populations at malaria endemic areas in Kenya, and reducing FBN30 expression by RNAi makes mosquitoes more susceptible to *P. berghei*. New results show that FBN30 is a secreted octamer that binds to both *P. berghei* and clinically circulating *P. falciparum* from malaria endemic areas in Kenya, but not laboratory *P. falciparum* strain NF54. Moreover, the natural genetic mutation (T to C) within FBN30 signal peptide, which changes the position 10 amino acid from phenylalanine to leucine, reduces protein expression by approximately half. This change is consistent to more susceptible *An. gambiae* to *P. falciparum* infection in the field. FBN30 in natural *An. gambiae* is proposed to work as a pathogen recognition molecule in inhibiting *P. falciparum* transmission in malaria endemic areas.

## Introduction

Mosquitoes transmit many human diseases such as malaria, dengue fever, and Zika. Due to absence of adaptive immunity, mosquitoes rely on innate immunity to mount a defense against these pathogens. Molecules that recognize these pathogens play important roles in initiating an innate immune response. Among them, the family of fibrinogen-related proteins (FREPs, also known as fibrinogen domain immunolectins or FBNs) is reportedly involved in innate immunity against malaria and other pathogens^1^. FBNs contain a conserved domain(FBG) of approximately 200 amino acids with some sequence similarity to the C-terminus of mammal fibrinogen β and γ chains^2^. The fibrinogen domains from insects share a great degree of similarity with 28% to 46% amino acid identity^3^.

In invertebrates, the function of FBNs is mainly for defense instead of blood coagulation^2, 4, 5^. FBNs have been identified from a wide range of species functioning as pattern-recognition receptors (PRRs) through the binding of carbohydrates on the outer membrane pathogens. Tachylectins 5A and 5B (TL5A and TL5B) are FBNs from the horseshoe crab, which specifically bind the subunit of chitin (N-acetylglucosamine) to mark invading pathogens for phagocytosis by hemocytes^6, 7^. FREPs identified from several tick species including OmFREP and Dorin M from *Ornithodoros moubata* and Ixoderin A and B from *Ixodes ricinus* also have the functions of self/non-self recognition and agglutination^8–10^. In addition, the FREPs from the snail, *Biomphalaria glabrata*, not only recognize pathogens but also show functional specialization of different pathogens^11^. FBN9 in mosquitoes works as a PRR activating the mosquitoes’ immune response against both Gram-negative and Gram-positive bacteria by direct interaction^12, 13^.

*Anopheles gambiae* is the major malaria vector in Africa. Due to the application of antimalarial drugs, insecticide-treated nets, and indoor insecticide spraying, deaths caused by malaria have considerably declined in recent years. Nonetheless approximately half a million people died from malaria last year, and most of them were under the age of five^14^. The natural interplay between mosquitoes and parasites could be used to eliminate parasites in mosquitoes and block malaria transmission. However, little is known about this interaction in natural settings. A recent genome wide association of *Plasmodium falciparum* infected mosquitoes in Kenya identified two FREPs, FREP1 and FBN30, which significantly affected parasite infection in mosquitoes^15^. Further studies showed that FREP1 mediates *Plasmodium* to invade mosquito midguts by anchoring ookinetes to the peritrophic matrix^16^. However, silencing FBN30 in *An. gambiae* increased *P. berghei* infection intensity in mosquitoes, supporting that FBN30 inhibits *Plasmodium* infection.

In this study, we determined that FBN30 is a secreted protein and forms an octamer. Using ELISA and immunofluorescence assays (IFA), we found that FBN30 is able to interact with *P. berghei* and clinically circulating *P. falciparum* isolates, but not with laboratory *P. falciparum* strain NF54. Moreover, we elucidated that the identified natural mutation within FBN30 signal peptide affects the expression efficiency of FBN30. Together, mosquito FBN30 is proposed to be a pathogen recognition molecule against clinically circulating *P. falciparum* infection in natural *An. gambiae* populations in malaria endemic areas in Kenya. This molecule could be used as a target by small molecules to reduce malaria burden.

## Results

### Recombinant FBN30 protein is secreted from insect cells and forms octamer

**Figure 1:**
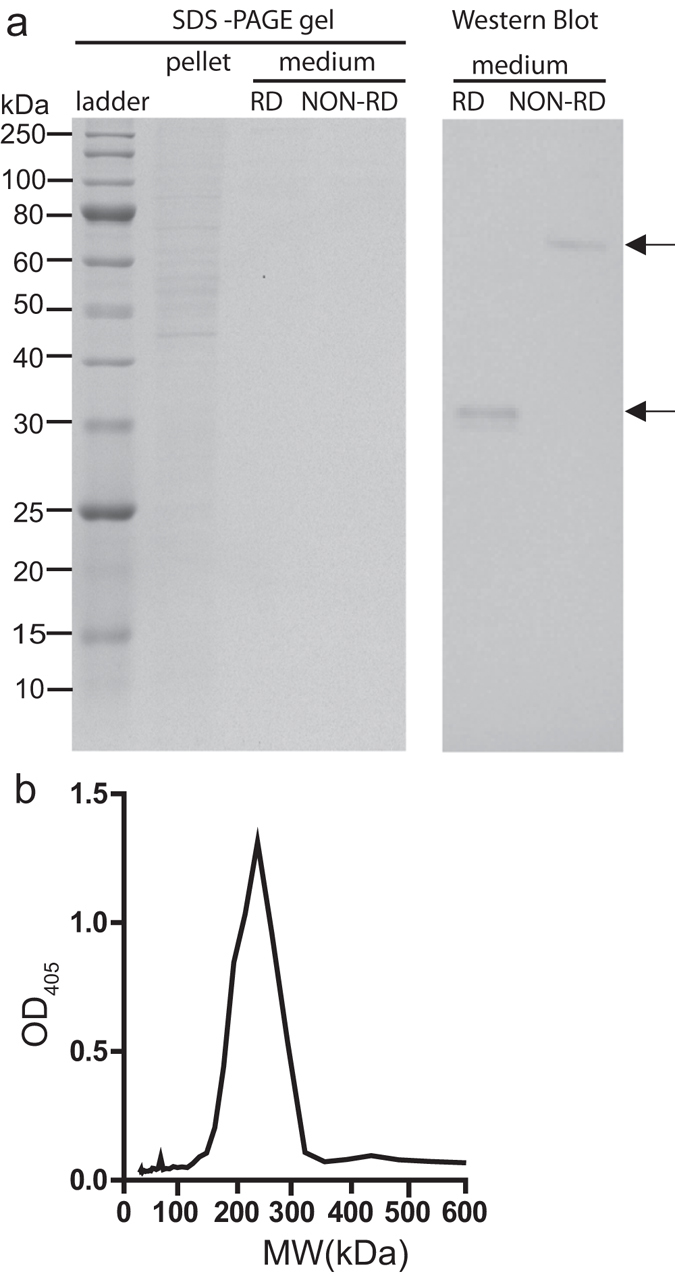
FBN30 is secreted from insect Hi5 cells and forms octamers. (**a**) The non-reducing (NON-RD) (without β-ME) and the reducing (RD) conditions of 12% SDS-PAGE analysis were performed. FBN30 was detected with anti-FBN30 antibody. A specific band with a molecular mass of ~ 33 kDa (bottom arrow labeled, corresponding to insect cell expressed recombinant FBN30 protein) under reducing condition was detected from the medium (right panel), indicating that the recombinant FBN30 is a secreted protein. Under non-reducing conditions, a specific band of ~66 kDa (top arrow labeled) was detected with anti-FBN30, suggesting that FBN30 forms a homodimer through disulfide bond. (**b)** The FBN30 protein from the concentrated medium was applied onto a size exclusion chromatography using a Superdex-200 increase column and the FBN30 in each eluted fraction was quantified with ELISA assays. The molecular mass of FBN30 ranged from 212–274 kDa, suggesting that the native FBN30 exists as an octamer, which is formed by four disulfide-bond linked homodimers.

FBN30 contains 280 amino acids. It includes a signal peptide and a coiled-coil domain at the N-terminus. A  FBG domain is present at the C-terminus. To determine the biochemical features of FBN30, *FBN30* coding region was cloned by PCR with specific primers (Table 1) from *An. gambiae*, inserted into plasmid pIB/V5-His. Since culturing High Five (Hi5) cells (derived from cabbage looper ovary cells) does not need serum, we used Hi5 cells to express recombinant protein to simplify the protein purification process. After a three-day post-transfection of Hi5 cells with the recombinant plasmid, the medium and cell pellets were collected by centrifugation and analyzed by SDS-PAGE and western blot. A specific band was detected in the culture medium under reducing condition by  western blot assay (Fig. 1a, right panel). The molecular weight of this protein was about 33 kDa, which matches the predicted size of the mature FBN30 protein without the signal peptide. This band was not detected in cell pellets (Fig. S1). Therefore, FBN30 was secreted from cells into the medium. Under non-reducing conditions, the 33 kDa band disappeared and a 66 kDa band was shown in the cell culture medium in the western blot assay (Fig. 1a). This result suggests that FBN30 subunits form a homodimer through a disulfide bond.

**Table 1:** PCR Primers.

Primer name	Primer sequence
Clone *FBN30(T)* variant into pIB/V5-His forward primer	5′-TCA*GAATTC*ACCATGCTGCTCGCAACAGTGTTCCTCGT G***T***CTGGTGCAGTGCA-3′
Clone *FBN30(C)* variant into pIB/V5-His forward primer	5′-TCA*GAATTC*ACCATGCTGCTCGCAACAGTGTTCCTCGT G***C***CTGGTGCAGTGCA-3′
Common reverse primer	5′-TT*CTAGAG*GTGCACTACGAAGCCGAAT-3′

Note: The italic and underlined sequences denote restriction recognition sites. The italic and bold sequences denote difference. The primers were synthesized through Integrated DNA Technologies Inc. (USA).

Next, we examined the quaternary structure of the insect cell- expressed FBN30 under native conditions. The concentrated medium containing insect cell-expressed recombinant FBN30 was subjected to size exclusion chromatography and the amount of FBN30 in each eluted fraction was quantified with enzyme-linked immunosorbent assay (ELISA). The calculated molecular weight of FBN30 based on the eluted FBN30 peak ranged from 212–274 kDa (Fig. 1b). Given that the observed molecular mass (~66 kDa) of the disulfide-bond linked FBN30 homodimer, this result indicates that the native FBN30 formed octamers of four disulfide-bond linked homodimers through non-covalent bonds.

### FBN30 protein binds to *P. berghei* infected red blood cells and ookinetes

**Figure 2:**
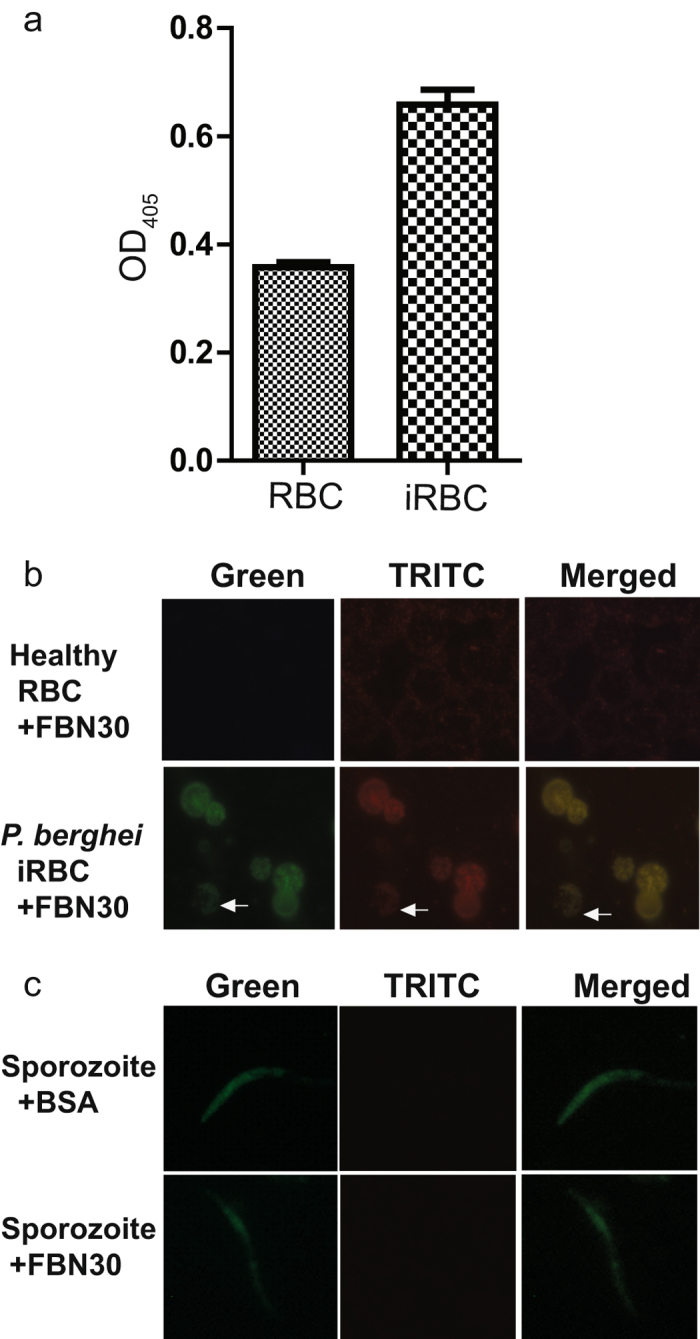
FBN30 binds to *P. berghei* infected red blood cells and ookinetes. (**a**) Interaction between the recombinant FBN30 protein and *P. berghei* iRBC by ELISA. The lysates of *P. berghei*-infected red blood cells (iRBC) and uninfected mouse red blood cells (RBC) were used to determine the interaction between FBN30 and *P. berghei* parasites. OD_405_ values and standard deviations were obtained from 3 replicates. The OD_405_ value in *P. berghei* iRBC was 1.86 folds of the uninfected mouse RBC, and the difference is significant (*p *< 0.01). (**b)** Interaction between FBN30 protein and *P. berghei* parasites determined by indirect immunofluorescence assays (IFA). Images in the first row represent non-infected mouse RBCs. Images in the second row represent mouse *P. berghei* iRBCs and ookinetes. Images in the third row obtained by merging first and second rows to show the co-localization of FBN30 and parasites. Arrows show the location of *P. berghei* gametocytes. (**c**) FBN30 does not bind to *P. berghei* sporozoites as determined by IFA. Images in the first row are the control group by replacing FBN30 with equal amount of BSA in IFA assays. The first and second columns show *P. berghei* parasites (green color) and FBN30 (red color) respectively. Merging column one and two generated the third column.

Since FBN30 inhibited *P. berghei* infection^15^, we examined the molecular mechanism of this inhibition. First, we used ELISA assays to investigate the interaction between the insect cell expressed recombinant FBN30 and lysates of *P. berghei* infected mouse red blood cells (iRBC) containing 5–10% gametocytes. The iRBC lysates were used to coat ELISA plates, followed by incubation with recombinant FBN30. The bound FBN30 was quantified by anti-FBN30 antibodies. An un-infected mouse RBC lysate was used as a control. Figure 2a shows that OD_405_ in *P. berghei* iRBC was 1.86 folds of the uninfected mouse RBC, suggesting that *P. berghei* iRBC retained significantly more FBN30 than un-infected mouse RBC (*p* < 0.01). Furthermore, we used IFA to examine the binding of FBN30 protein to *P*. *berghei* iRBC, ookinetes, and sporozoites. *P. berghei* iRBCs and ookinetes were fixed on glass slides with 4% paraformaldehyde, and probed with the insect cell-expressed recombinant FBN30 protein. Anti-FBN30 antibody and fluorescence conjugated secondary antibodies were used sequentially to determine the bound FBN30 proteins. GFP-transgenic *P. berghei*-infected cells and ookinetes emit green color under fluorescence. Results showed that the recombinant FBN30 protein (red) co-localized with *P. berghei*-infected cells (Fig. 2b, 2^nd^ row) and ookinetes (Fig. 2b, 2^nd^ row, arrow indicated), while the control group did not show any red fluorescence (Fig. 2b, 1^st^ row). We also determined whether there is any interaction between *P. berghei* sporozoites and FBN30 by IFA. The results were negative, showing no interaction between sporozoites and FBN30 (Fig. 2c). Together, these results support that FBN30 binds to blood-stage *P. berghei* and ookinetes, but not sporozoites.

### FBN30 protein binds to clinically circulating *P. falciparum* isolates

**Figure 3:**
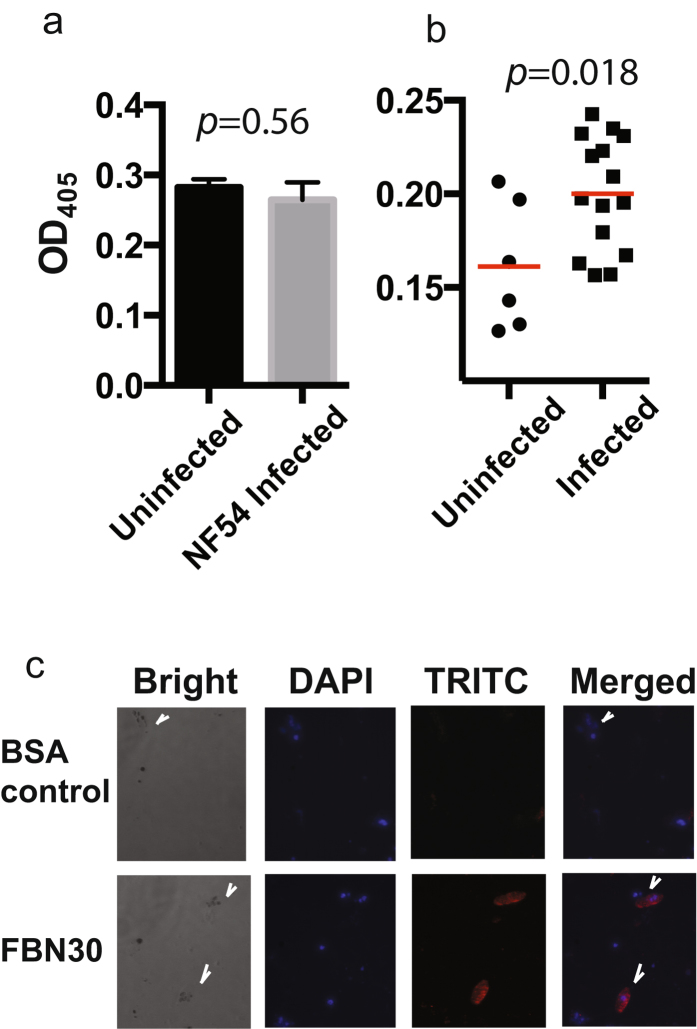
FBN30 interacts with clinically circulating *Plasmodium falciparum* isolates. (**a**) FBN30 does not bind to laboratory *P. falciparum* strain NF54. There is no significant difference between uninfected RBC and NF54 infected RBC in ELISA assays. (**b**) The ELISA data show that significantly more FBN30 bound to blood lysates from the malaria infected patients (15 individuals) than that from the uninfected human subjects (6 individuals) (*p* = *0.018*). (**c**) FBN30 interacts with the cultured wild type *P. falciparum* ookinetes determined by IFA. Images in the first row are the control group in which the anti-FBN30 was replaced with BSA. Arrows show the location of ookinetes.

Next, we determined whether FBN30 also binds to human malaria pathogen *P. falciparum*. First, we used NF54, a laboratory *P. falciparum* strain in ELISA assays. Five independent uninfected human RBC samples were used to culture NF54 to get gametocytes and ookinetes as we described previously^16^. These uninfected human RBCs and 15–17-day cultured NF54 cells (containing 20–30% asexual stage parasites, 5–10% gametocytes, and many merazoites) were lysed and used to coat ELISA plates, followed by incubation with insect cell-expressed FBN30. Surprisingly, anti-FBN30 antibodies did not detect significantly more FBN30 bound to NF54 infected cell lysates than uninfected human RBC lysates (*p *= 0.54) (Fig. 3a), suggesting that FBN30 does not interact with NF54 parasites.

Because direct association studies found that FBN30 was related to *P. falciparum* transmission to mosquitoes in malaria endemic areas^15^, we hypothesized that FBN30 might interact with clinically circulating *P. falciparum* isolates from Kenya. We collected blood samples from 15 *P. falciparum* infected patients and six local uninfected persons at Kisumu, Kenya under the same settings. The blood samples were washed with PBS and cells were lysed in lysis buffer. The ELISA data showed that the blood lysates from the malaria infected patients bound significantly more FBN30 compared to that from the uninfected subjects (Fig. 3b, *p* = *0.018*).

Furthermore, an IFA approach was used to demonstrate the direct interaction between FBN30 and clinically circulating *P. falciparum* ookinetes. Fortunately, we successfully cultured two wild malaria isolates under laboratory conditions. Using the IFA method as we described previously^16, 17^, results clearly showed that FBN30 co-localized with wild type *P. falciparum* ookinetes (Fig. 3c). After replacing FBN30 with BSA in the control, the binding signals disappeared.

### The mutation within FBN30 signal peptide changes its expression efficiency

**Figure 4:**
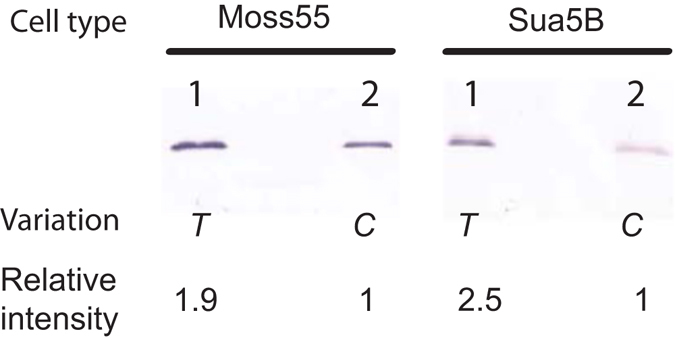
Expression efficiency of two variants of *FBN30* in mosquito cell lines. Moss55 cells(left) and Sua5B cells(right) were the mosquito cells transfected with vector. After transfection assays with the same amount of recombinant plasmids containing either *FBN30* (*T*) or (*C*), the western blot assays detected one specific band corresponding to the recombinant FBN30 protein. The band intensity was measured with Image J software. The expression ratios of *FBN30* (*T*) to (*C*) were 1.9 and 2.5 folds in Moss 55 and Sua5B respectively.

A genetic variation within FBN30 was reported to be associated with *P. falciparum* infection intensity in *An. gambiae*. This natural mutation from thymine to cytosine at the position 28 of *FBN30* CDS changes the position 10 amino acid from phenylalanine (Phe) to leucine (Leu). This mutation renders *An. gambiae* mosquitoes more susceptible to *P. falciparum*. Here, we investigate why these two variants alter mosquitoes’ susceptibility to parasites. Since this mutation is within FBN30 signal peptide and FBN30 is a secreted protein as we demonstrated in the previous section, we hypothesized that this mutation affects the protein expression efficiency. We amplified the *FBN30* gene from *An. gambiae* with a pair of specific primers (Table 1), and cloned the complete FBN30 gene into pIB/V5-His plasmid. As expected, sequencing determined that the gene in the plasmid had a thymine at position 28. Then, another pair of primers (Table 1) that mutate thymine to cytosine were used to clone the gene. Sequencing data confirmed that the coding sequences were identical except at position 28. To get FBN30 expression and secretion in mosquito cells (Hi5 cell was not originated from mosquitoes), these two variants of *FBN30* in plasmid pIB/V5-His were used to transfect two mosquito cell lines (Moss55 and Sua5B). After incubation, the cell culture supernatant was analyzed with western blot assays by an anti-His monoclonal antibody. After transfection with the same amount of plasmids containing either *FBN30* (*T*) or (*C*), one specific band corresponding to FBN30 protein was detected (Fig. 4 Lane 1 or 2, Supplemental Fig. S2). The expression ratios of *FBN30* (*T*) to (*C*) were measured and calculated as 1.9 and 2.5 fold in Moss 55 and Sua5B cells respectively. These assays were repeated twice and showed consistent results. It is worth noting that higher expression efficiency of FBN30 with T at position 28 (phenylalanine) in mosquito cells correlated to more resistance to clinically circulating *P. falciparum* infection in wild *An. gambiae* populations^15^.

## Discussion

Non-self-recognition by FREPs functioning as pathogen recognition receptors (PRRs) to initiate innate immunity against pathogens has been described in several invertebrate species^4^. We have previously reported that a natural genetic variation within FBN30 was associated with *P. falciparum* infection in mosquitoes and silencing of FBN30 significantly increased *P. berghei* infection intensity in *An. gambiae*, indicating FBN30 plays a role in the defense against *Plasmodium* infection in *An. gambiae* ^15^. This study demonstrates that *An. gambiae* FBN30 could specifically bind to *P. berghei* and clinically circulating *P. falciparum*.

We examined the biochemical characters of FBN30, demonstrating that FBN30 is a secreted octamer. The type of PRRs, which includes FREPs, complement receptors, collectins, ficolins, and peptidoglycan recognition proteins (PGRs) tend to be secreted to tissues where they become active^2^. Also, their functional forms usually exist as oligomers, which can increase binding strength compared with the monomeric form^18, 19^. For instance, FBN9 forms a homodimer through disulfide bonds and the structure is considered to improve their pathogen-binding capability^12^. A lectin from horseshoe crab (TL-5A) forms a similar homo-tetrameric structure^6, 7^. The size exclusion chromatography analysis demonstrates that the native FBN30 also forms a dimer-tetramer structure with four disulfide-bond linked homodimer units by non-covalent bonds. Similar structures have been identified in human PAX5-PML protein^20^ and human guanylate-binding protein 1^21^. Apparently, FBN30 shares some common biochemical features with other pathogen recognition molecules.

Surprisingly, FBN30 interacts with clinically circulating *P. falciparum* isolates in Kenya but not with laboratory strain NF54. The capability of FBN30 to recognize and discriminate the infected cells from the uninfected cells supports its role as a PRR molecule. The ligands of the PRRs, also called as pathogen-associated molecular patterns (PAMPs), have been identified in some FREPs. Among them, some lectin-like FREPs are demonstrated to bind to carbohydrates. For instance, TL-5A interacts with a sugar containing acetyl group such as N-acetyl-glucosamine (GlcNAc)^6^, which perfectly fits in the active binding site, a hydrophobic funnel surrounded by aromatic side chains in the  FBG domain^22^. Similar structures have been identified in other FREPs, such as FIBCD1 and ficolins^23–25^. Nevertheless, it is unknown what molecules FBN30 binds to. It was a striking that FBN30 does not bind to laboratory *P. falciparum* isolate NF54, suggesting mutations in NF54 change FBN30 binding ligands. Therefore, caution needs to be taken in the use of NF54 strain as a laboratory model before the knowledge is applied to the field. Meanwhile, our newly established isolates from Kenya could be a more relevant resource for malaria research communities.

FBN30 also binds to *P. berghei*, a rodent malaria pathogen that is distantly related to *P. falciparum*, suggesting that FBN30 might be a broad-spectrum PRR against human malaria pathogens. FBN30 prevents *Plasmodium* from developing in mosquito midguts. However, FBN30 does not bind sporozoites, suggesting that FBN30 unlikely affects the migration of sporozoites from midguts to salivary glands or transmission of sporozoites from mosquito salivary glands to humans.

A previous publication identified a nonsynonymous mutation (C/T) at *FBN30* position 28 that is associated with field *P. falciparum* infection intensity in natural *An. gambiae* populations. Here, we determine how this natural genetic variation within the FBN30 signal peptide changes the susceptibility of wild *An. gambiae* to clinically circulating *P. falciparum*. Variations in length and composition of signal peptide sequences that change secretory efficiency of a mature protein have been reported in other organisms including humans^17, 26, 27^. For example, the insertion/deletion polymorphisms of apolipoprotein B within its signal peptide are associated with plasma glucose levels in Europeans and Mexican Americans^28^. Similarly, a single nucleotide polymorphism (SNP) in the signal peptide sequence can significantly affect the expression of a gene and interferes with its physiological functions, as noted in the human neuropeptide Y (T1128C). This non-synonymous nucleotide mutation causes an amino acid change from Leucine to Proline (Leu7Pro) in the signal peptide, which was associated with increased body mass in Swedish populations^29^. Our data showed that FBN30 (*T*) has approximately 2-fold higher expression than *FBN30* (*C*) in mosquito cell lines. The varying expression of FBN30 is due to the change from Phe to Leu at the amino acid position 10 in the predicted signal peptide. The SNP of *FBN30* (*T28C*) results in one amino acid change in the signal peptide (Phe10Leu) and dramatically reduced the secreted FBN30 expression in mosquito cells. The impact of this mutation is reflected in the susceptibility of natural *An. gambiae* populations to *P. falciparum* infection^15^.

In summary, FBN30 inhibits parasite development in mosquitoes. It is a secreted octamer, and binds to clinically circulating *P. falciparum* in natural *An. gambiae* through specific recognition. The natural genetic mutation within FBN30 signal peptide changes FBN30 expression level and alter the susceptibility of mosquitoes to *P. falciparum*. This study elucidates a molecular basis for mosquito-malaria interaction in nature.

## Materials and Methods

### Use of experimental animals and human participants

Human subjects were used to obtain *P. falciparum* infected blood and health blood. The experiments were carried out according to US National Institute of Health guidelines and Kenya government regulations, and followed Kenya Medical Research Institute approved IRB protocol (NON-SSC PROTOCOL NO. 213). Written, informed consent was provided by every study participant and/or legal guardian. All animal experiments were carried out in strict accordance with the recommendations in the Guide for the Care and Use of Laboratory Animals of the US National Institute of Health. The Institutional Animal Care and Use Committee at the University of Oklahoma approved the procedure (R15-012).

### Rearing *An. gambiae* mosquitoes

*An. gambiae* G3 strain was maintained at 27 °C, 80% humidity with a 12-hour light-dark cycle. Larvae were fed with KOI fish food supplements (0.1 mg per larvae per day) and adult mosquitoes were maintained with 8% sucrose and fed with mouse blood for egg production.

### Generating polyclonal antibody

FBN30 contains 280 amino acids with a signal peptide. *FBN30* encoding mature protein was PCR-cloned from *An. gambiae* using specific primers of 5′-ACAT*GCATGC*ATGCTGCTCGCAACAGTGTTC-3′ and 5′-AA*CTGCAG*CTACGGTGCACTACGAAGCC-3′, and inserted it into plasmid pQE30. The Ni-column purified FBN30 protein expressed in *E. coli* was used to generate antibodies in rabbits. This polyclonal antibody could recognize FBN30 specifically and was used as a tool to detect FBN30 protein in this report.

### Expression of the recombinant FBN30 protein in insect cells

Two variants of *FBN30* (*T/C*) were cloned into vector of pIB/V5-His (Life Technology, Grand Island, NY) by RT-PCR with gene specific primers (Table 1) from *An. gambiae* mosquitoes. The plasmid DNAs extracted with endotoxin-free plasmid preparation kits (Sigma-Aldrich, St. Louis, MO) were used in transient expression with Hi5 insect expression system. Transfection assays were performed using Cellfectin® Reagent (Life Technology) according to the manufacturer’s protocol. Cells were seeded in a 25 cm^2^ flask and formed an approximately 20% monolayer 24 hr prior to transfection. A total of 10 µg plasmid DNA was used to transfect into Hi5 cells in a flask. Next, the cells and medium containing the expressed FNB30 were collected 48 hr post-transfection and trace amount of Hi5 cells in the medium were removed by centrifugation at 300 x g for 5 min. To compare the expression of two variants in mosquito cells, 2.5 µg plasmid of either pIB-FBN30-C or pIB-FBN30-T were transfected into Moss 55 and Sua5B respectively in a 6-well plate using Lipofectamine® LTX Reagent with PLUS™ Reagent (Life Technology) following the company’s manual. The medium containing FBN30 proteins were collected after 48 hr. The FBN30 expression in the medium was analyzed with western blot assays using anti-His monoclonal antibody. The expression intensity was measured based on western blot by the software of Image J (developed by Wayne Rasband).

### Reducing, non-reducing SDS-PAGE, and gel filtration analysis

To determine whether the disulfide bonds exist in FBN30, the non-reducing (without β-ME) and reducing conditions of 12% SDS-PAGE gel were performed and the expression of FBN30 in the cellular pellet and medium were detected by using anti-FBN30 antibodies. To check the molecular weight of the native FBN30, which may form oligomers, proteins were further separated using size exclusion chromatography. Samples were applied onto a Superdex 200 10/300 GL size exclusion column (GE, Fairfield, CT) on an AKTA Pure FPLC system (GE). The column was pre-calibrated to generate a standard curve with a set of proteins differing in size (Gel Filtration LMW Calibration Kit, GE). The chromatography elution buffer used was phosphate buffered saline (PBS, 3 mM sodium phosphate dibasic, 1mM potassium phosphate monobasic, 155 mM sodium chloride, pH 7.4, Life Technology) and the sample volumes were set at 500 µL. The elution was carried out using isocratic elution at a flow rate of 0.4 mL/min and the fractions were collected 100 µL per fraction. FBN30 protein in each elution fraction was measured by using polyclonal anti-FBN30 antibodies in ELISA.

### Preparation of *P. berghei* infected red blood cells and sporozoites

*P. berghei* (ANKA strain) expressing green fluorescent protein (GFP) was used to infect mice through i.p. injection. The parasitemia was checked every other day by Giemsa staining of thin blood smears. When parasitemia reached 10%, gametocytes were induced 2–3 days before infection by treating mice with 60 mg phenylhydrazine hydrochloride (Santa Cruz Biotechnology, Dallas, Texas) per Kg body weight (4mg/mL, dissolved in PBS). The infected blood was collected, and incubated to generate ookinetes^16^.

To isolate sporozoites, around 100 two-day-old female mosquitoes were fed with a *P. berghei* infected mouse. The fully engorged mosquitoes were kept in an insectory at 20 °C for 18–20 days. The centrifugation method together with Ozaki tubes was used to purify sporozoites^30^. Briefly, infected mosquitoes were rinsed in 70% ethanol for 1 min and washed with the dissection medium (DMEM medium, Life Technologies) supplemented with 0.1% normal mouse serum and 2X Penicillin-Streptomycin (Life Technologies). Salivary glands were dissected and transferred to an Ozaki tube containing 100 µL dissection medium, and then centrifuged at 6,700 xg for 1.5 min. Purified sporozoites were collected as a pellet from the bottom of the 1.5 mL microcentrifuge tube and were re-suspended in 50 µL dissection medium. The density of the purified sporozoites was counted with a hemocytometer under light microscope.

### Preparation of cultured wild strain *P.falciparum* isolates

The *P. falciparum* infected human blood was collected from patients in highland areas near Kisumu, western Kenya. The parasites were cultured in a 6-well cell culture plate with each well containing 200 µL fresh O^+^ human blood (red blood cells, RBCs) and 4.8 mL incomplete RPMI1640 medium containing supplements of 2.5 mg/mL lipid enriched bovine serum albumin (Albumax II, Life Technologies) and 12.5 µg/mL hypoxanthine at 37 °C in a candlejar. Medium was replaced daily until typical ring-stage parasites were observed by the blood smear with Giemasa staining. Afterwards, heat-inactivated human AB^+^ type serum (Interstate blood bank, Memphis) was added gradually (1% increment per day until 10% was reached) into the culture medium, and simultaneously, bovine serum albumin was progressively decreased by 0.25 mg/mL per day. Parasites were then maintained and ookinetes were generated using the same protocols as NF54 strain as described previously^16^. The parasitemia or gametocytemia was checked regularly with Gemisa Staining.

### Binding assays between FBN30 and *Plasmodium* parasites by ELISA

*P. berghei*-infected mouse  RBC and uninfected mouse  RBC were collected, washed three times with PBS, and re-suspended in PBS containing 0.2% Tween-20 (PBST). The lysates were prepared by ultrasonication of cells for six time with 10 sec on and 50 sec off on ice, and then centrifuged at 8, 000 x g for 5 min to remove intact cells and cellular insoluble aggregates. The proteins in supernatants were used for ELISA assays. The protein concentration was measured using the Bradford method. A 96-well plate (Brand, Wertheim, Germany) was coated with 2 mg/mL lysates and incubated overnight at 4 °C. The next day, each well was then incubated with the following solutions: 200 µL blocking buffer (2 mg/mL BSA in PBS) for 1.5 hr, 100 µL recombinant FBN30 protein (100 µg/mL) at RT for 1hr, 100 µL of anti-FBN30 antibody (1:2,000 dilution with blocking buffer) for 1 hr at RT and 100 µL of alkaline phosphatase-conjugated anti-rabbit IgG (1:20, 000 dilution with blocking buffer) for 45 min at RT. The wells were washed with PBST (PBS with 0.2% tween-20) three times between each incubation. At the end, the wells were developed with 100 µL of pNPP solution (Sigma-Aldrich, St. Luis, MO) until the colors appeared. Finally, the absorbance at OD_405_ was measured.

### Indirect immunofluorescence assay (IFA)

To test interaction between FBN30 and parasites, standard IFA was performed as described previously^16, 17^. The uninfected mouse RBC, the *P. berghei* iRBC or sporozoites were deposited on glass slides (Fisher Scientific) and immediately fixed in 4% paraformaldehyde in PBS at RT for 30 min, which preserved intact (non-permeabilized) cell membranes. The glass slide was then sequentially incubated as the following: 10 mM glycine in PBS for 20 min, blocking buffer (2mg/mL BSA in PBS) for 2 hr, anti-FBN30 antibody (1:1,000 dilution in blocking buffer) for 2 hr, enhancer (Alexa Fluor ® 594 Goat Anti-Mouse SFX kit, Life Technologies) for 30 min, and secondary antibody (Alexa Fluor ® 594 Goat Anti-rabbit Antibody, 1:1,000 dilution in blocking buffer, Life Technologies) for 30 min. The slides were washed three times with blocking buffer for 3 min between each incubation. At the end, the glass slide was rinsed with distilled water for 20 sec and mounted with vectashield mounting media (Vector Laboratories, Burlingame, CA). The cells and parasites were examined under fluorescence microscopy after incubation for at least 2 hr in darkness. The negative control of FBN30-sporozoite interaction used an equal amount of BSA to replace FBN30 protein. In the other experiment, the cultured infected red blood cells of the wild strain *P.  falciparum* were deposited onto glass slides and the assays were conducted following the methods mentioned above.

## Electronic supplementary material

Supplemental Figures
