# Supplementary material for: FBN30 in wild Anophelesgambiae functions as a pathogen recognition molecule against clinically circulating Plasmodiumfalciparum in malaria endemic areas in Kenya
Source: Sci Rep. 2017 Aug 17;7:8577. doi: 10.1038/s41598-017-09017-3 (PMC5561218; doi:10.1038/s41598-017-09017-3)
Supplement: Supplementary file 1 — Supplemental Figures [file 41598_2017_9017_MOESM1_ESM.pdf]

FBN30 in wild *An. gambiae* functions as a pathogen recognition molecule against clinically circulating *P. falciparum* in malaria endemic areas in Kenya

Guodong Niu[1], Genwei Zhang[2], Caio Franca[2], Yingjun Cui[1], Stephen Munga[3], Yaw Afrane[3], and Jun Li[1,†]

[1] Department of Biological Sciences, Biomolecular Sciences Institute, Florida International University, Miami, Florida, USA 33199

[2] Department of Chemistry and Biochemistry, University of Oklahoma, Norman, Oklahoma, USA 73019

[3] Centre for Global Health Research, Kenya Medical Research Institute, Kisumu, Kenya

[†] Corresponding author:

Dr. Jun Li

Department of Biological Sciences

Florida International University

11200 SW 8th Street, Miami, USA

Tel: 305-348-7618

Email: lij@fiu.edu

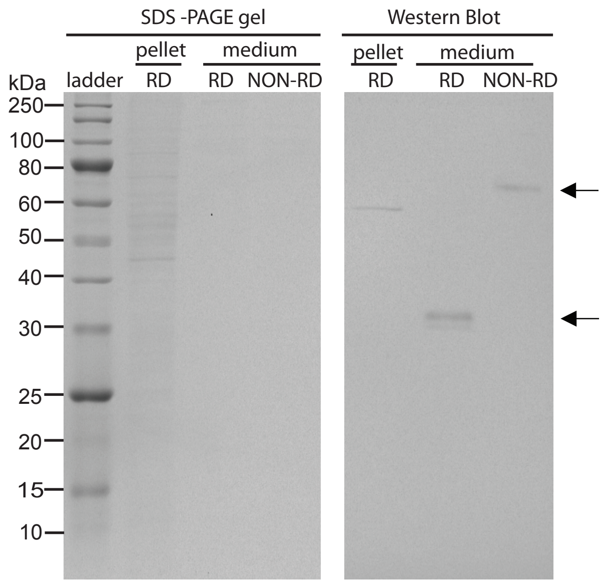

Figure S1: FBN30 is secreted from insect Hi5 cells.

The non-reducing (NON-RD) (without β-ME) and the reducing (RD) conditions of 12% SDS-PAGE analysis were performed. FBN30 was detected with anti-FBN30 antibody. A specific band with a molecular mass of ~ 33 kDa (bottom arrow labeled, corresponding to insect cell expressed recombinant FBN30 protein) under reducing condition was detected from the medium (right panel), indicating that the recombinant FBN30 is a secreted protein. Under non-reducing conditions, a specific band of ~ 66 kDa (top arrow labeled) was detected with anti-FBN30, suggesting that FBN30 forms a homodimer through disulfide bond. A nonspecific band with molecular weight of 58 kDa was found in cell pellet of western blot. Comparing the much higher protein concentration in cell pellet sample than medium shown in Coomassie blue stained SDS-PAGE (left panel), the nonspecific binding is very weak.

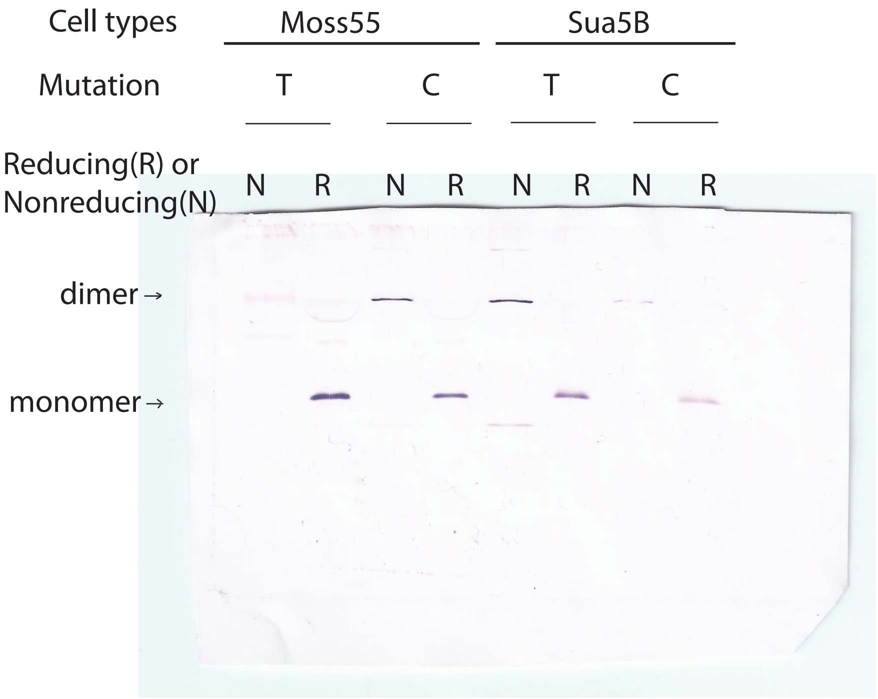

Figure S2: Original picture of western blot assays of *FBN30* gene expression.

Two plasmids with nucleotide thymine or cytosine with *FBN30* gene (position 28) were transfect into mosquito cell lines (Moss55 and Sua5B) and expressed. The expressed FBN30 in culture medium was separated by 12% SDS-PAGE at reducing (with 2-mercaptoethanol) or non-reducing conditions, transferred into membrane, and detected by anti-His antibody.
